# Editorial: Insights in thrombosis and haemostasis: from a biological, clinical and genetic perspective

**DOI:** 10.3389/fcvm.2025.1645473

**Published:** 2025-07-08

**Authors:** Cristina Tudoran, Raluca Dumache, Gabor Erdoes, Irina Andra Tache, Florica Voita-Mekeres

**Affiliations:** ^1^ Department VII, Internal Medicine II, Discipline of Cardiology, University of Medicine and Pharmacy “Victor Babes” Timisoara, Timisoara, Romania; ^2^ Center of Molecular Research in Nephrology and Vascular Disease, University of Medicine and Pharmacy “Victor Babes” Timisoara, Timisoara, Romania; ^3^ Cardiology Department, County Emergency Hospital “Pius Brinzeu”, Timisoara, Romania; ^4^ Neuroscience Department, “Victor Babes” University of Medicine and Pharmacy, Timisoara, Romania; ^5^ Department of Anesthesiology and Pain Medicine, Inselspital, University Hospital Bern, University of Bern, Bern, Switzerland; ^6^ Department of Automatic Control and Systems Engineering, National University of Science and Technology POLITEHNICA Bucharest, Bucharest, Romania; ^7^ Department of Morphological Disciplines, Faculty of Medicine and Pharmacy, University of Oradea, Oradea, Romania; ^8^ Department of Psychiatry, County Clinical Emergency Hospital Bihor, Oradea, Romania

**Keywords:** cardiovascular disease, thrombosis, haemostasis, pulmonary embolism, coronary artery disease

**Editorial on the Research Topic**
Insights in thrombosis and haemostasis: from a biological, clinical and genetic perspective

Thrombosis and haemostasis are the two essential physiological properties of blood. Their primary purpose is to prevent bleeding, but even small changes at the molecular level may alter this process, leading to complex pathophysiological alterations that affect both arteries and veins, subsequently resulting in morphological and functional changes in the tributary territories. A detailed clinical evaluation may sometimes indicate the location and severity of embolism/thrombosis, but in most situations, multiple investigations are necessary to establish an accurate diagnosis.

“Insights in Thrombosis and Haemostasis: from a Biological, Clinical and Genetic Perspective” debates aspects of deep vein thrombosis (DVT), starting with several genetic alterations and culminating with peculiar clinical presentations. A second section refers to laboratory investigations capable of revealing the underlying pathways of atherosclerosis and platelet aggregation.

In “Insights in Thrombosis and Haemostasis: from a Biological, Clinical and Genetic Perspective”, we present several research studies that have focused on defining the mechanisms responsible for DVT, aiming to improve its prevention. Some studies observed that an elevated blood urea nitrogen and creatinine ratio (BCR) level was independently linked with an increased risk of in-hospital mortality among critically ill patients diagnosed with venous thrombo-embolism (VTE). Given its widespread availability and ease of measurement, BCR could be a valuable tool for risk stratification and prognostic prediction in VTE patients. Other scientists determined that aberrant gene expression, at the level of MicroRNAs, usually 21–23 nucleotides long, can impact biological functions and influence the development and evolution of cardiovascular and venous diseases. The epigenetic modification of forkhead box protein 3 (FOXP3) at the posttranscriptional level, might be the key trigger leading to the down-regulation of FOXP3 expression in patients with DVT. Overexpressed miR-6132 reduced FOXP3 expression and aggravated DVT formation, while miR-6132 knockdown increased FOXP3 expression and alleviated DVT formation. The microRNAs regulating the expression of FOXP3 might represent novel targets for future DVT treatment options.

Subsequently, we discuss the connection between the development of DVT and physiological conditions (pregnancy), and some predisposing pathologies (especially spinal cord injury). Pregnant women with DVT pose various challenges for diagnosis and treatment. To decrease the risk for both mother and child, we focused on the early detection, diagnosis, and treatment of pregnancy-associated venous thromboembolism by providing an overview of radiological diagnostic techniques in this particular situation. Among the pathologies associated with DVT, cancer, and spinal cord injuries are associated with the worst prognosis for these patients. By screening 232 patients with spinal cord injury and cervical fracture, we observed a high rate of DVT exacerbation. The worsening of existing DVT or the development of new DVT was favoured by older age, ASIA score A-B, time from injury to surgery, surgery time, and blood loss. Surprisingly, several cases of DVT that occurred in younger men (under 40 years old) have been described. Although all experts consider older age a risk factor for DVT, in recent years, an increase in the incidence of lower limb DVT has been detected even in young adults, under 40 years old. Several particularities have been observed: male sex, the frequent involvement of the inferior vena cava, which could at least partially explain the higher incidence of DVT recurrence.

One of the main challenges in patients with DVT is adequate treatment, which starts with nursing care and ends with oral anticoagulants and/or surgical interventions. Nursing care should focus on emphasizing comprehensive preoperative and postoperative evaluations to optimize patient outcomes. These evaluations facilitate tailored treatment plans, crucial for managing the complex needs of DVT patients. Regarding the long-term anticoagulation, most practitioners prefer direct oral anticoagulants, which offer a safer profile and simplified management compared to traditional therapies. Mechanical interventions are also an alternative (balloon angioplasty and venous stenting) as they can improve immediate and long-term vascular function in acute cases. Furthermore, the use of image-guided techniques is essential for enhancing the accuracy and safety of these interventions. The combined use of systemic thrombolysis with Tenecteplase and anticoagulation may be a treatment option for acute extensive portal venous system thrombosis (PVST) in the future. There are no recent studies that examine the association between antifactor-Xa (AFXa) based heparin monitoring and clinical outcomes in the setting of cerebral venous thrombosis (CVT). Although monitoring UFH based on AFXa values may be practical, reaching target AFXa levels within 24 h of hospitalization may not necessarily be prognostic.

In the second part of our topic, we discuss the underlying pathophysiological mechanisms relevant to the occurrence of coronary artery disease (CAD). Inflammation, oxidative stress, and the activation of the sympathetic nervous system form an interconnected network, with each component influencing the others and creating a vicious circle. To better evaluate patients scheduled for bypass surgery, it would be beneficial to develop a scoring system including biological markers indicating inflammation and oxidative stress. This could help us to identify patients that require a more aggressive approach to lower inflammation, oxidative stress, and modulate the sympathetic nervous system. Some studies suggested that oxidative stress and inflammation can be worsened during coronary artery bypass grafting (CABG). Remote ischemic preconditioning (RIPC), a non-invasive approach to protect the heart and other organs from ischemia and reperfusion injury, may improve the outcome of these patients. Patients undergoing RIPC before CABG had reduced plasma levels of Lectin-like oxidized low-density lipoprotein (LDL) receptor-1 (LOX-1) and increasing total values of Superoxide dismutase-1 (SOD-1) after surgery.

Platelet endocytosis was first mentioned in the medical literature in the 1980s when the existence of active internal systems in platelets was first identified. Transmission electron microscopy enabled the observation of platelets internalizing small particles (such as fibrinogen, IgG, and albumin) into membranous structures through a phagocytosis-like mechanism. Platelet endocytosis also has a role in haemostasis and various pathophysiological processes. Platelet endocytosis may be influenced by viral infections, such as the severe acute respiratory syndrome coronavirus 2 (SARS-CoV-2). Platelets in patients with COVID-19 exhibited hyperactivity, but a newer theory suggests that the SARS-CoV-2 virus could be ingested by platelets, as it was demonstrated that the virus could penetrate the platelet surface in the intracellular platelets. Other data supporting this hypothesis are the fact that mRNA traces of SARS-CoV-2 were detected in isolated platelets, and virions could be observed within platelet sections.

Despite the direct effects of the virus on the human body, soon after the development of the first vaccine and its extensive use, several adverse effects were reported. Although rare, one of the most severe ones was clotting and thrombocytopenia events, followed by subsequent coagulation abnormalities, more frequently encountered after adenovirus-DNA-based vaccines (Janssen/Johnson & Johnson and Astra-Zeneca vaccines). A new pathology was defined, the Vaccine-Induced Immune Thrombotic Thrombocytopenia (VITT), characterized by unusual cerebral and splanchnic venous thrombosis, and circulating autoantibodies directed against anti-platelet factor 4 (PF4). VITT has a very low incidence and does not affect the overall benefit of immunization; however, if left untreated, it can be debilitating or even fatal.

This topic is the result of team work, with the combined effort of the editors, with the help of the contributors and of the entire editorial team from Frontiers in Cardiovascular Medicine.

